# Novel Core–Shell Nanostructure of *ε*-Poly-L-lysine and Polyamide-6 Polymers for Reusable and Durable Antimicrobial Function

**DOI:** 10.3390/polym17233195

**Published:** 2025-11-30

**Authors:** Saloni Purandare, Rui Li, Chunhui Xiang, Guowen Song

**Affiliations:** 1 Textile Technology Program, Gaston College, Belmont, NC 28012, USA; purandare.saloni@gaston.edu; 2 Department of Apparel, Events, and Hospitality Management, Iowa State University of Science and Technology, Ames, IA 50011, USA

**Keywords:** ε-poly-L-lysine, polyamide-6, nanofiber, coaxial electrospinning, core–shell nanofiber, antimicrobial, non-toxic, reusable antimicrobial, stable antimicrobial

## Abstract

Antimicrobial function in protective and medical textiles is an essential safety feature since textiles can become breeding grounds for microorganisms. Ideally, the antimicrobial function in textiles should be non-toxic, stable, and durable. This study explores a core–shell nanofiber with a core of the cationic biopolymer ε-poly-L-lysine (PL) and shell of structurally similar and biocompatible polyamide-6 (PA). The core–shell structure is expected to have a more stable antimicrobial function than its monolithic counterpart. Further, thermal crosslinking is expected to prevent rapid diffusion of the water-soluble PL. Therefore, this study establishes a comparison between a monolithic (control), a core–shell (CS), and a thermally crosslinked core–shell (CL-CS) nanofiber of PL and PA. Morphological analysis confirmed the successful generation of the core–shell nanofibers. All the samples exhibited hydrophilic behavior and antimicrobial function. However, the control sample showcased significantly reduced zones of inhibition in antimicrobial testing with 21 days of bacterial exposure (1.027 ± 0.072 cm^2^), as compared to 24 h bacterial exposure (1.347 ± 0.151 cm^2^). On the other hand, the zones of inhibition for 24 h vs. 21 days for CS (1.265 ± 0.042 cm^2^ vs. 1.052 ± 0.235 cm^2^) and CL-CS (1.128 ± 0.161 cm^2^ vs. 1.106 ± 0.047 cm^2^) showed no significant differences. Therefore, the core–shell structure allowed for sustainable and durable antimicrobial action. Lastly, the CL-CS sample exhibited reusable antimicrobial function owing to the core–shell structure paired with thermal crosslinking. This study showcases a fiber system with non-toxic, durable, and reusable antimicrobial function. This study builds grounds for the development and multifaceted holistic characterization of safe, stable, and scalable antimicrobial textiles.

## 1. Introduction

Textiles can turn into a host for microorganisms, posing a threat to the safety and health of wearers while diminishing the useability of the textile. Antimicrobial function in textiles prevents bacterial transfer from the environment to the wearer and vice versa, thus assisting in the health and hygiene of the wearer’s surroundings [1,2]. Therefore, it is important to include antimicrobial functions in textiles, especially in the case of protective and medical textiles. Nanofibers are fibers with diameters less than 1000 nm. Along with other functional applications, nanofibers are now largely being explored for antimicrobial textile applications due to their high loading capacity for antimicrobial ingredients, large surface areas, spinnability for a wide range of polymers, and easy manipulation of structural features [3].

A variety of active antimicrobial ingredients can be utilized for the generation of antimicrobial nanofibers. The selection of the ingredients should be such that there is no toxicity or health hazard to the wearer [2]. Metal and metal oxides have efficient antimicrobial properties and are largely used to impart antimicrobial functions to nanofibers. However, higher concentrations of these active ingredients might be associated with health hazards [4]. Further, commonly used synthetic antimicrobial agents such as triclosan and Quaternary Ammonium Salts (QAS) have also been associated with toxicity towards wearers and the environment. This highlights the need for natural and non-toxic antimicrobial agents [5].

Several research advancements are currently focused on utilizing naturally occurring antimicrobial ingredients in textiles. Some examples of natural agents being explored for their antimicrobial properties in nanofibers are natamycin, green tea, and rosemary extract [6]; aloe vera gel [7]; and photocatalytic vitamin K [8]. Furthermore, biopolymers such as pectin (citrus peels), alginate, and chitosan are also being researched for non-toxic antimicrobial properties [9]. One such biopolymer with inherent antimicrobial properties is ε-poly-L-lysine (PL). This cationic biopolymer is produced by Streptomyces albulus bacteria in sources like sugar and glycerol [10,11]. PL showcases efficient antimicrobial activity against Gram-positive and Gram-negative bacteria via electrostatic absorption, is biocompatible, and has been found to selectively target bacterial over mammalian cells [10,11,12,13,14]. PL has been successfully loaded into various nanofiber systems such as those of chitosan [15,16], gelatin [14], polyhydroxybutyrate [17], Persian gum–polyethylene oxide [18], cellulose [19], and polyamide-6 [20] for antimicrobial functions in applications such as food packaging, wound dressing, and medical textiles.

Along with the non-toxic nature of the antimicrobial agent, it is also essential that its antimicrobial function is preserved throughout the use cycle of textiles. Reusability and stability of antimicrobial function in a textile is a key characteristic to allow for its large-scale adoption as well as to contain the growing amount of medical textile waste. Despite being a vital characteristic, only a few studies address the durability of antimicrobial agents in nanofibers [3,21,22].

Durable antimicrobial function in textiles is possible through sustained release of antimicrobial agents for its long-term stability and by protecting the antimicrobial agent from diminishing efficiency during use. Chemical manipulation and crosslinking are one route to improving the adhesion of antimicrobial agents in fiber systems. Another route is morphological variation of fibers to better trap antimicrobial agents in fibers to allow for their sustained delivery. Such kinds of morphological variation in nanofibers can be developed via coaxial electrospinning. Electrospinning is a popular method of developing nanofibers by electrifying a viscoelastic solution to generate a charged jet which, upon stretching and elongation, results in a nanofiber [23]. A traditional electrospinning setup involves the use of a single nozzle spinneret, resulting in a monolithic nanofiber structure. Coaxial electrospinning, on the other hand, involves the use of a specialized spinneret with two nozzles for supplying two different spinning solutions such as a single jet [24,25]. A core–shell fiber structure via coaxial electrospinning allows for encapsulation of antimicrobial agents within the core protected by a polymeric shell [26]. This structure improves the lifespan of antimicrobial agents as compared to its monolithic nanofiber counterparts by preventing burst release of the agent and allowing its sustained delivery, while also protecting the antimicrobial agent from any possible environmental factors that contribute towards antimicrobial action deterioration [26,27,28].

Polyamide-6 (PA) is a commonly utilized textile polymer in medical and protective textiles due to its high mechanical performance in wet and dry conditions, biocompatibility, chemical resistance, and resistance to bodily fluids [29]. Despite its advantages, PA exhibits biofilm tendency by allowing bacterial growth on its surface, limiting its applicability in medical and protective textiles [30]. Therefore, the utility of PA can be enhanced by incorporating a stable and durable antimicrobial function. This study aims to propose a PA nanofiber system that has a stable, durable, and non-toxic antimicrobial function and is scalable, intended for functional textile applications. To accomplish this, this study explores the development and characterization of a core–shell fiber system with a core of cationic PL biopolymer encapsulated by a structurally similar PA shell. This novel combination of a core–shell structure is expected to possess efficient antimicrobial performance that allows for its reusability and stability of its antimicrobial function.

## 2. Methods

### 2.1. Materials

ε-Poly-L-lysine (PL) polymer (Mw = ~3500–4700 Da, purity ≥ 95%) was purchased from Zhengzhou Bainafo Bioengineering Co., Ltd. (Zhengzhou, China). Polyamide-6 (PA) polymer (Mw = ~10,000 Da) and formic acid (88%) were purchased from Sigma-Aldrich (St. Louis, MO, USA). Nutrient Agar and *Escherichia coli* (K–12) bacterial culture were purchased from Carolina Biological Supply Company (Burlington, NC, USA).

### 2.2. Electrospinning Solution

A monolithic fiber composition was used as the control sample for this study in order to establish a comparison between monolithic vs core–shell nanostructures of PA and PL polymers. The spinning solution for the control sample was prepared by dissolving PA (30% *w*/*v* of formic acid) and PL (40% *w*/*w* of PA) polymers in formic acid [20]. The core–shell nanofiber (hereon referred as CS) was spun using two spinning solutions which were prepared separately. The spinning solution for the core consisted of PL (30% *w*/*v* of formic acid) in formic acid while the spinning solution for the shell consisted of PA (30% *w*/*v* of formic acid) in formic acid. All the spinning solutions were freshly prepared in batches of 6 mL and stirred overnight to ensure homogeneity.

### 2.3. Electrospinning

Nanofiber membranes were developed using an electrospinning technique. The spinning solutions were supplied to the spinneret needle at a constant rate using a syringe pump (Harvard Apparatus, Holliston, MA, USA). The tip of the needle was connected to a fixed voltage supply using a DC power supply instrument (Gamma High Voltage Research, Ormond Beach, FL, USA). The nanofibers were collected at a fixed distance from the needle on a grounded collector.

The control sample was developed using a needle with a single nozzle (18 gauge). As for the CS sample, a custom needle (Rame-Hart Instrument Co., Succasunna, NJ, USA) with a core (22 gauge) and a shell (18 gauge) nozzle was used. The spinning parameters were 1 mL/h feed rate, 20 KV voltage supply, 12 cm spinning distance, and 4 h of collection time. Nanomembranes of different spinning compositions were developed in a randomized sequence to average out the effect of possible extraneous variables.

### 2.4. Thermal Bonding of Core–Shell Nanofibers

Thermal bonding is a form of physical crosslinking that can be used to bond and stabilize fibers within non-woven nanomembranes. Thermal bonding in nanofiber membranes can be utilized for improved mechanical performance and reduced water solubility [31,32,33]. PL is water-soluble and thus, to allow reusable and stable antimicrobial function, the CS nanomembranes were crosslinked at 80 °C for 6 h under vacuum. The crosslinked CS nanomembranes are hereon referred to as CL-CS.

### 2.5. Morphological Analysis

Transmission Electron Microscopy (TEM, Philips, Morgagni 268, Cambridge, MA, USA) with an accelerating voltage of 20 KV was utilized to confirm the formation of core–shell nanofiber structure. The TEM test samples were prepared by directly spinning CS nanofibers onto copper grids for a few seconds. Then, the diameter and surface features of the control, CS, and CL-CS nanofibers were analyzed using a Field Emission Scanning Electron Microscope (FESEM) (FEI Quanta 250, ThermoFisher Scientific, Waltham, MA, USA). FESEM test samples were kept in vacuum overnight prior to imaging. The nanofiber diameters from the TEM and FESEM images were measured using Image J software (Version 1.54, National Institute of Health, Bethesda, MD, USA) and are reported as averages of 50 representative fibers.

### 2.6. Chemical Characterization

The functional groups and chemical interaction within the nanomembranes were observed using Fourier Transform Infrared (FTIR) spectrometer (Perkin-Elmer Frontier FTIR, Waltham, MA, USA). For all the test samples, a FTIR spectrum was obtained in the range of 800–4000 cm^−1^ with 4 cm^−1^ wavenumber resolution, and each measurement consisted of 32 scans.

Further, X-Ray Photoelectron Spectroscopy (XPS) was conducted on all the samples with a Kratos Amicus XPS system (Kratos Analytical Ltd., Manchester, UK) to characterize the surface chemical compositions of the nanomembranes.

### 2.7. Thermal Analysis

Thermal analysis was conducted using a differential scanning calorimeter (PerkinElmer DSC 4000, Shelton, CT, USA) and thermal gravimetric analysis (TGA 5500, TA Instruments, New Castle, DE, USA). During DSC analysis each sample was heated to 400 °C at a heating rate of 20 °C min^−1^. As for TGA analysis, the nanofiber films were heated from 25 °C to 400 °C at a heating rate of 10 °C min^−1^. All the samples for thermal analysis weighed approximately 10 mg and the entire process was performed under nitrogen atmosphere (40 mL min^−1^ flow rate).

### 2.8. Water Contact Angle

The hydrophilicity of the nanomembranes was evaluated by measuring the static water contact angle using a video-based drop shape analyzer (OCA 25, Data Physics GmbH, Regensburg, Germany) and built-in SCA20 software (V.4.5.20). The analysis was conducted as per the ASTM-D7334-08 sessile drop method [34]. Testing was conducted in triplicate on separate nanomembranes for all the test samples.

### 2.9. Antimicrobial Analysis

The antimicrobial properties of all the samples were evaluated as per the AATCC-147 zone inhibition method in triplicate on separate nanomembranes [35]. Nutrient agar plates were prepared and seeded with 1 mL *Escherichia coli* inoculums containing approximately 4 × 10^6^ Colony Forming Units (CFU)/mL. Test samples were then plated in contact with the bacteria for 24 h at 37 °C. The test samples were observed for zones of inhibition post-incubation.

### 2.10. Antimicrobial Performance upon Long-Term Exposure to Bacteria

The testing was conducted as per AATCC-147, as described above, in triplicate on separate nanomembranes [35]. In this case, the test samples were incubated with the bacteria for a period of 21 days, and the zones of inhibition were observed.

### 2.11. Reusability of Antimicrobial Function

The antimicrobial function of developed nanomembranes was tested for reusability. Antimicrobial testing was conducted as per AATCC-147, and at the end of each antimicrobial test, the test sample was washed with 95% ethanol for ~30 s and with deionized water for ~30 s and then dried in a 60 °C oven [35,36]. Three cycles of reusability were performed, and three separate nanomembranes were included from each sample type for each of the test cycles.

### 2.12. Statistical Analysis

The results in this study were analyzed with SPSS software (version 29.0; IBM Corp., Armonk, NY, USA) via one-way analysis of variance (ANOVA) and Tukey statistical tests. All results are expressed as mean ± standard deviation and *p*-value < 0.05 was considered significant. Different letters in the superscripts in the tabular data indicate significant differences (*p* < 0.05).

## 3. Results and Discussion

### 3.1. Morphological Analysis of Nanofibers

**Figure 1:**
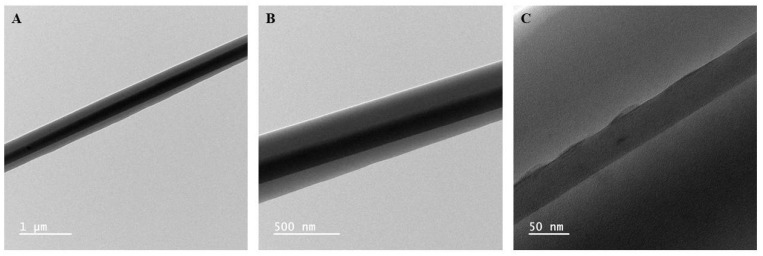
TEM images of CS nanofibers imaged at (**A**) 10,000×, (**B**) 25,000×, and (**C**) 150,000× showing the core and shell structures.

TEM imaging of CS samples was conducted to confirm the formation of core and shell structure within the nanofibers. As seen in Figure 1, the distinct contrast in the brightness between the core and shell phases of the nanofiber allows us to confirm the presence of the two phases. Therefore, based on the TEM images, the spinning solution composition and spinning parameters used successfully translated into a core–shell nanofiber morphology. Based on the TEM images, Table 1 showcases the average core diameter and shell thickness of the core–shell nanofibers.

**Figure 2:**
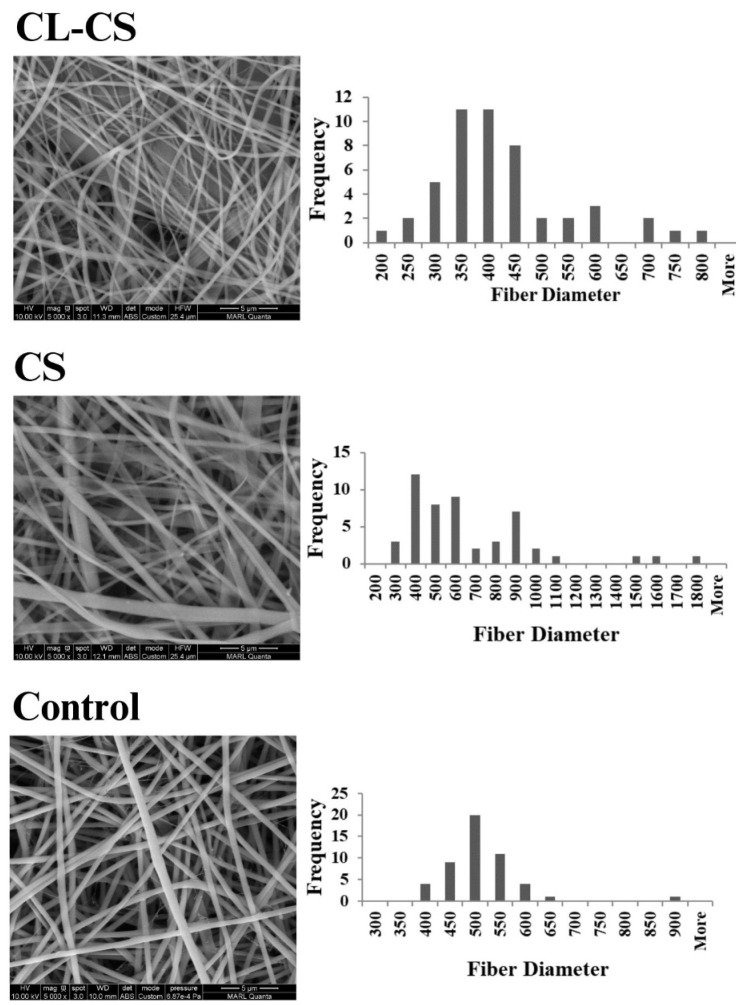
FESEM images of developed nanomembranes along with their respective fiber diameter distribution.

FESEM imaging was further conducted for all the samples to observe the surface features and to measure fiber diameters. Figure 2 shows the FESEM images along with fiber diameter frequency distribution graphs for each sample. The average diameters of the nanofibers are listed in Table 2. The FESEM images indicate the formation of continuous nanofibers for all the three samples, thus validating the spinning parameters. Nanoribbon structure is visible for some of the nanofibers in the CS and CL-CS samples. Nanoribbon structure can be formed when there is a hindrance in solvent dissipation from core of spun jet due to the solidification of outer layer while the interior is still filled with undissipated solvent. Such a solvent-rich jet collapses as a flat nanofiber upon reaching the collector. This phenomenon is common with thick viscous jets [37,38]. In this case, it can be assumed that, upon dissipation of solvent molecules from the surface, the viscous spinning solution of comparatively high-molecular-weight PA shell hindered further dissipation of the solvent-rich core made of lower-molecular-weight PL, thus forming the flat nanofibers. Further, the CL-CS image demonstrates bundles of bonded fibers, thus confirming that the exposure of nonwoven fiber web to temperatures higher than the carrier polymer’s glass-transition temperature facilitates inter-fiber cohesion [31].

### 3.2. Chemical Characterization of Nanomembranes

**Figure 3:**
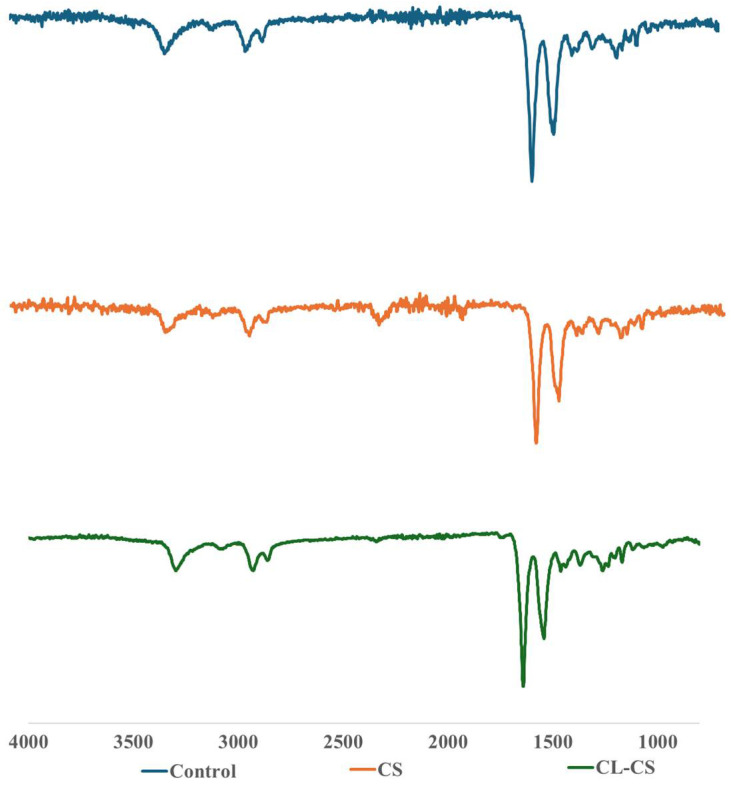
FTIR analysis of nanomembranes showcasing the observed chemical composition.

The elemental composition of all the nanomembranes was analyzed using FTIR analysis (see Figure 3). The analysis demonstrated the characteristic peaks of both PA and PL polymers in all the samples, thus confirming their presence. The peaks characteristic to PL are ~1642 cm^−1^ and ~1542 cm^−1^, indicating C=O and N–H bending (amide II) stretching vibration, respectively [16,39,40]. Peaks at ~3300 cm^−1^ signify hydrogen-bonded N–H group. And those at ~2850 cm^−1^ and ~2930 cm^−1^ signify CH_2_ stretching. These peaks are characteristic of PA [41]. A red shift is predicted for amide A in case of replacement of the H-bonds with water to H-bonds with amide [42]. A slight shift was observed from 3307 cm^−1^ for the CS sample to 3295 cm^−1^ for the CL-CS sample. This red shift thus could be due to the possible replacement of amide–water hydrogen bonds with amide–amide hydrogen bonds during dehydrothermal crosslinking [43].

**Figure 4:**
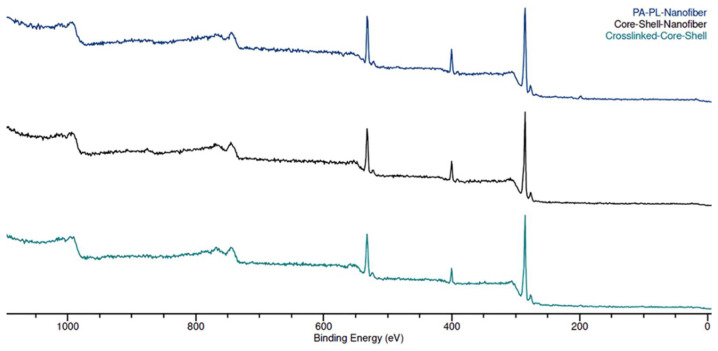
XPS wide scan of nanomembranes showcasing the surface-level analysis of chemical composition.

The XPS wide scan spectrum for all the nanomembranes showed the presence of carbon (C), oxygen (O), and nitrogen (N) (see Figure 4). PL has a higher N/O ratio in its chemical structure as compared to PA, which can be used to track the presence of PL on fiber surface [44]. The control sample exhibited a higher N/O ratio as compared to the CS and CL-CS samples (see Table 3). This is because the control sample is a mix of PA and PL in a monolithic fiber system and is expected to have the presence of both polymers on the surface. However, with the core–shell structure trapping PL within the core, only the nitrogen group from PA is expected on the surface. Therefore, the N/O ratio from XPS analysis further validates the different fiber structures of the samples.

### 3.3. Thermal Analysis of Nanomembranes 

**Figure 5:**
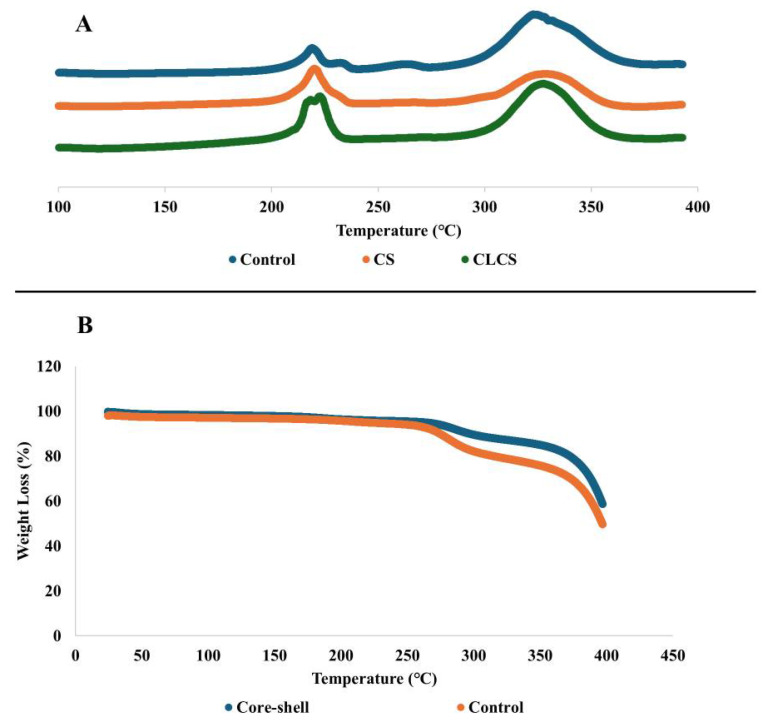
Thermal analysis of nanomembranes via (**A**) DSC analysis and (**B**) TGA.

The DSC analysis of nanomembranes demonstrated endothermic peaks representing the melting behavior of the samples (see Figure 5A). The onset of melting for control was at 208.30 °C and a second endothermic peak was observed at 291.16 °C [20]. For the CS and CL-CS samples, the melting onset was at 210.89 °C and 210.27 °C, and a second endothermic peak was observed at 312.13 °C and 309.04 °C, respectively. Multiple melting peaks can be an indication of secondary crystallization (melting–recrystallization–remelting) [45,46]. However, the absence of crystallization exotherm between the two melting peaks could be an indication of two separate phases generated during electrospinning [47]. The melting point of PA nanomembranes is 207.05 °C [20], while the melting peaks of PL powder are at 306 °C and 321 °C [39]. Therefore, the two endothermic peaks in the samples analyzed can be said to belong to the two polymers. The phase separation in the control can be accounted for by the increased PL concentration in the fiber system [20], possibly resulting in distinct phases. However, there is also an indication of possible interactions between the two polymers in the control sample since the melting onset, particularly for PL, is different from that of individual polymers. And as for the core–shell nanofibers, the presence of two melting peaks indicates the presence of two different phases of the core and shell structure [48].

TGA of the control and CS nanomembranes is shown in Figure 5B. For both the samples, a small weight loss of around 5% was observed until ~250 °C, around 20% weight loss until ~340 °C, and a more dramatic weight loss thereafter. Both the samples retained around 50% of their weight at 400 °C.

### 3.4. Static Water Contact Angle

**Figure 6:**
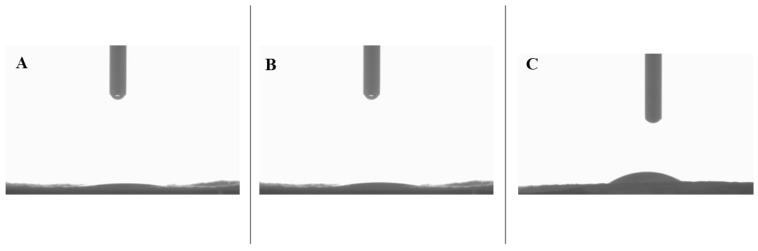
Hydrophilicity analysis of nanomembranes via static water contact angle of (**A**) control, (**B**) CS, and (**C**) CL-CS nanomembranes.

The hydrophilicity of all the samples was analyzed by measuring static water contact angles, as shown in Figure 6. For both control and CS nanomembranes, the water contact angle between the droplet and nanomembrane became zero within the first 10 s of contact. This indicates highly hydrophilic behavior from the measured nanomembranes. PA nanomembranes are slightly hydrophilic with a water contact angle of 82.44 ± 9.72° at the 10 s mark [20]. The control sample in this study is a monolithic nanofiber with a mix of PA and PL polymers. Therefore, the inclusion of PL with PA results in improved hydrophilicity due to the amino groups in PL’s structure that have affinity towards water molecules [14,20]. As for the CS nanomembrane, the highly hydrophilic PL core is responsible for its superhydrophilicity, either because PL readily absorbs water droplets that channel through PA or because of the presence of a few PL molecules close to/on the surface [49,50,51]. Lastly, CL-CS nanomembrane exhibited a decrement in hydrophilicity with a static contact angle of 23.13 ± 1.91° at the 10 s mark. Thermal crosslinking is expected to introduce additional intermolecular crosslinks by replacing moisture via condensation due to the high temperature [33]. Thus, the slightly reduced hydrophilic behavior in the CL-CS nanomembranes could possibly be an indication of successful crosslinking and reduced water solubility in PL.

Overall, all the nanomembranes are hydrophilic and hydrophilicity is expected to facilitate the contact-induced antimicrobial action of PL. The cationic amino group in PL attacks bacterial anionic cell membrane upon contact, thereby disrupting its life cycle [10,52]. Hydrophilic surface properties allow absorption and rapid spreading of contaminated fluids, which helps with the contact-induced bacterial inactivation via electrostatic absorption [53]. Hydrophilicity is also desirable for user comfort and in applications such as the inner layer of a face mask, wound dressing, and sanitation.

### 3.5. Antimicrobial Behavior of Nanomembranes

**Figure 7:**
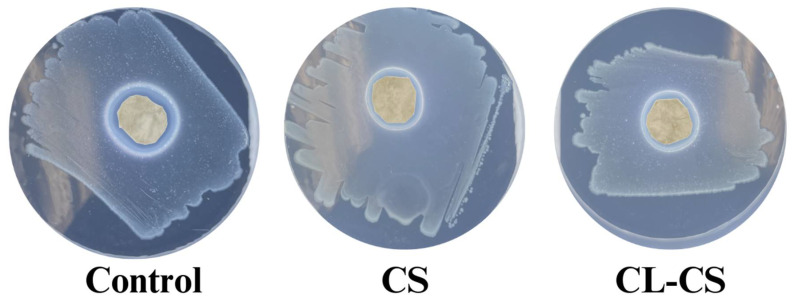
Observed zones of inhibition during antimicrobial testing of nanomembranes after 24 h of incubation with bacteria.

The antimicrobial performance of the nanomembranes was tested as per the AATCC-147 zone inhibition method. After 24 h of incubation with the bacteria, all the samples (control, CS, and CL-CS) exhibited antimicrobial action through evident zones of inhibition (see Figure 7 and Table 4). The antimicrobial behavior observed in all the samples could be tied to the presence of cationic PL in each of them, which is known to exhibit contact-induced antimicrobial action through electrostatic absorption [10,12].

**Figure 8:**
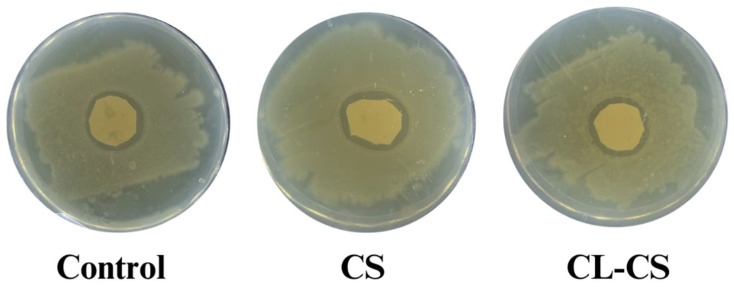
Observed zones of inhibition during antimicrobial testing of nanomembranes after 21 days of incubation with bacteria.

Cationic polymers such as PL can experience deteriorating antimicrobial function over time when there is exhaustion of cationic charges upon accumulation of cell debris on surfaces [54]. Therefore, it is crucial to test the antimicrobial performance of nanomembranes against bacteria for a prolonged period. Such testing also showcases the sustained delivery of antimicrobial agents and allows for the demonstration of clinical relevance [13,22,55]. This study placed nanomembranes with bacterial culture for a period of 21 days under favorable bacterial growth conditions to test whether there would be sustained delivery and active cationic action after prolonged bacterial exposure. All the samples (control, CS, CL-CS) exhibited zones of inhibition after 21 days of incubation, thus ensuring antimicrobial activity despite prolonged bacterial exposure (see Figure 8 and Table 5). The control sample exhibited significantly reduced antimicrobial activity after 21 days of incubation in comparison to its antimicrobial activity after 24 h of incubation (*p* < 0.05). However, the CS and CL-CS samples showed no significant differences between their antimicrobial activities after 24 h and 21 days of incubation (see Table 4 and Table 5). This indicates that a core–shell nanostructure can allow for sustainable and durable antimicrobial function.

**Figure 9:**
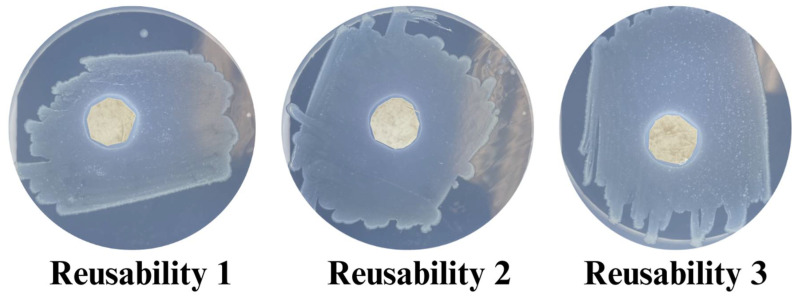
Observed zones of inhibition during antimicrobial testing of CL-CS nanomembranes for three cycles of reusability.

The reusability of antimicrobial performance was tested by rinsing and drying the samples between antimicrobial test cycles. The reusability of antimicrobial function was tested for three cycles. It was found that the CL-CS sample sustained all three cycles and continued to exhibit antimicrobial function. However, the antimicrobial functions of the other two samples (control and CS) did not sustain reusability testing. The reason for the lack of reusable antimicrobial function in the control and CS could be due to the water-soluble nature of PL [56], along with the swelling tendency and relative hydrophilicity of PA [20], causing the PL to rapidly diffuse from the nanofiber system in the presence of water [57]. Physical crosslinking via thermal bonding could have improved the water resistance of water-soluble polymers [32,33,58], while the core–shell structure provided a polymeric protective shell for the core. Therefore, the thermally bonded CL-CS nanomembranes preserved PL within the PA shell by preventing its rapid diffusion when exposed to rinsing, thereby allowing for reusable antimicrobial function for all three rinsing cycles (see Figure 9 and Table 6).

## 4. Conclusions

Antimicrobial textiles are crucial to ensure the health and safety of the wearer, especially in medical settings. Two criteria that remain important in terms of antimicrobial properties in textiles are non-toxic antimicrobial agents and stable antimicrobial function that remains effective during use of textile [3,5]. Electrospun nanofibers have various advantageous properties such as a large surface area to volume ratio, high loading capacity, and ease of spinnability. Therefore, today, electrospun nanofibers are largely being explored in functional textile applications including antimicrobial textiles. Coaxial electrospinning is used to generate morphological variations in conventional monolithic electrospun nanofibers. Core–shell nanofibers via coaxial electrospinning can generate nanofibers with antimicrobial cores protected by polymeric shells, thereby allowing the sustained delivery of the antimicrobial core [26,27,28].

PL is a naturally occurring biopolymer that exhibits broad spectrum non-toxic antimicrobial performance owing to its cationic chemical structure [10,11]. This study explores the development of a core–shell fiber system by using PL as the core and structurally similar biocompatible PA as the shell (CS). A monolithic control sample of PL and PA was included for comparative analysis. Further, a thermally crosslinked core–shell sample (CL-CS) was also developed to support its reusable and durable antimicrobial properties by preventing rapid diffusion of the water-soluble PL [32,33].

Morphological analysis conducted via FESEM and TEM provided evidence of the creation of continuous nanofibers with core and shell phases. The elemental composition of the nanomembranes was analyzed via FTIR. The analysis exhibited characteristic peaks of PA and PL for all samples. The FTIR analysis also demonstrated a slight red shift for the amide A peak in CL-CS compared to the CS sample. This shift can be tied to the replacement of amide–water hydrogen bonds with amide–amide hydrogen bonds during dehydrothermal crosslinking [42,43]. XPS analysis indicated a higher N/O ratio for the control sample as compared to the CS and CL-CS samples. This demonstrates the presence of both the polymers on the surface of monolithic nanofibers and the absence of PL on the surface for core–shell structure. The static water contact angle test exhibited the hydrophilic behavior of all the samples as a result of the inherently hydrophilic PL being part of the fiber systems. Hydrophilicity is desirable for user comfort while benefiting the contact-induced antimicrobial action. All the samples (control, CS, and CL-CS) exhibited antimicrobial function by displaying zones of inhibition when incubated with bacteria for 24 h and 21 days. Antimicrobial function in the samples was due to the presence of cationic PL in the fibers. Unlike the control sample, the CS and CL-CS samples showed no significant differences between their antimicrobial activity after 24 h and 21 days of incubation, thus confirming that the core–shell structure allows for sustainable and durable antimicrobial action. Lastly, the CL-CS sample exhibited reusable antimicrobial function as opposed to the other two samples owing to its core–shell structure paired with thermal crosslinking, which prevented rapid diffusion of PL from the fiber system.

This study thus demonstrates the generation of a fiber system with non-toxic, stable, durable, and reusable antimicrobial function, achieved via structural variation and inclusion of natural antimicrobial agent. The developed fiber systems can be explored for medical textile applications such as the inner layer of face masks, wound dressing, and sanitation. This study hopes to build grounds for further exploration of non-toxic fiber systems that are capable of satisfying the multi-faceted requirements of antimicrobial textiles to ensure their practical applicability.

## Figures and Tables

**Table 1:** Average core diameter and shell thickness of core–shell nanofibers.

Average Core Diameter (nm)	Average Shell Thickness (nm)
196.79 ± 27.63	114.48 ± 34.14

**Table 2:** Average fiber diameters of nanofibers.

Sample	Average Diameter (Microns)
Control	0.49 ± 0.07
CS	0.62 ± 0.32
CL-CS	0.40 ± 0.12

**Table 3:** N/O ratio of nanomembranes from XPS analysis.

Sample	N/O Ratio
Control	0.750
CS	0.610
CL-CS	0.430

**Table 4:** Antimicrobial activity of nanomembranes after 24 h of incubation.

Sample	Diameter of Zone of Inhibition (cm^2^)
Control	1.347 ± 0.151 ^a^
CS	1.265 ± 0.042 ^a^
CL-CS	1.128 ± 0.161 ^a^

**Table 5:** Antimicrobial activity of nanomembranes after 21 days of incubation.

Sample	Diameter of Zone of Inhibition (cm^2^)
Control	1.027 ± 0.072 ^a^
CS	1.052 ± 0.235 ^a^
CL-CS	1.106 ± 0.047 ^a^

**Table 6:** Antimicrobial activity of CL-CS nanomembranes before and after reusability testing.

Antimicrobial Test		Diameter of Zone of Inhibition (cm^2^)
Before Reusability Tests		1.128 ± 0.161 ^a^
After Reusability Tests	Cycle 1	1.009 ± 0.119 ^a^
	Cycle 2	0.963 ± 0.043 ^a^
	Cycle 3	0.941 ± 0.048 ^a^

## Data Availability

The original contributions presented in this study are included in the article; further inquiries can be directed to the corresponding authors.
